# Immunization with the immunodominant *Helicobacter suis* urease subunit B induces partial protection against *H. suis* infection in a mouse model

**DOI:** 10.1186/1297-9716-43-72

**Published:** 2012-10-26

**Authors:** Miet Vermoote, Katleen Van Steendam, Bram Flahou, Annemieke Smet, Frank Pasmans, Pieter Glibert, Richard Ducatelle, Dieter Deforce, Freddy Haesebrouck

**Affiliations:** 1 Department of Pathology, Bacteriology and Avian Diseases, Faculty of Veterinary Medicine, Ghent University, Merelbeke, Belgium; 2 Laboratory for Pharmaceutical Biotechnology, Ghent University, Ghent, Belgium

## Abstract

*Helicobacter* (*H.*) *suis* is a porcine and human gastric pathogen. Previous studies in mice showed that an *H. suis* infection does not result in protective immunity, whereas immunization with *H. suis* whole-cell lysate (lysate) protects against a subsequent experimental infection. Therefore, two-dimensional gel electrophoresis of *H. suis* proteins was performed followed by immunoblotting with pooled sera from *H. suis*- infected mice or mice immunized with lysate. Weak reactivity against *H. suis* proteins was observed in post-infection sera. Sera from lysate-immunized mice, however, showed immunoreactivity against a total of 19 protein spots which were identified using LC-MS/MS. The *H. suis* urease subunit B (UreB) showed most pronounced reactivity against sera from lysate-immunized mice and was not detected with sera from infected mice. None of the pooled sera detected *H. suis* neutrophil-activating protein A (NapA). The protective efficacy of intranasal vaccination of BALB/c mice with *H. suis* UreB and NapA, both recombinantly expressed in *Escherichia coli* (rUreB and rNapA, respectively), was compared with that of *H. suis* lysate. All vaccines contained choleratoxin as adjuvant. Immunization of mice with rUreB and lysate induced a significant reduction of *H. suis* colonization compared to non-vaccinated *H. suis*-infected controls, whereas rNapA had no significant protective effect. Probably, a combination of local Th1 and Th17 responses, complemented by antibody responses play a role in the protective immunity against *H. suis* infections.

## Introduction

*Helicobacter* (*H.*) *suis* is a world-wide spread pathogen, mainly colonizing pigs. An infection with this Gram-negative bacterium has been associated with ulcers of the gastric non-glandular mucosa [1,2] and causes gastritis and decreased daily weight gain [3] in pigs. *H. suis* is also the most prevalent non-*Helicobacter pylori Helicobacter* species in humans suffering from gastric disorders [2] and pigs may serve as a source of *H. suis* infections for humans [2,4]. Control of *H. suis* infections by antibiotic-based therapy is not recommended partly due to an increased risk of developing acquired antimicrobial resistance in *H. suis* strains and in bacteria belonging to the normal porcine microbiota [5]. Immunization against *H. suis* may therefore represent a valuable alternative. Up to now, however, few studies have dealt with vaccination against this porcine and zoonotic pathogen.

Previous studies in a mouse model showed that an *H. suis* infection does not result in protective immunity, whereas vaccination based on homologous (*H. suis*) or heterologous (*H. bizzozeronii* or *H. cynogastricus*) whole-cell lysate induced a reduction or even complete clearance of gastric colonization with *H. suis*[6]. However, the use of this type of vaccines has drawbacks, including the laborious in vitro culture of *H. suis*, which results in difficulties to produce sufficient antigen. Also, whole-cell lysates may contain both protective antigens and antigens suppressing protection [7]. An effective subunit vaccine might be a useful alternative for control of *H. suis* infections. Immunoproteomics is an appropriate approach for rapid identification of candidate proteins for vaccination and has been applied to study and develop subunit vaccines for a wide range of pathogens [8].

It was the aim of the present study to select *H. suis* proteins which might induce protective immunity against *H. suis* infection. Therefore, *H. suis* proteins recognized by sera of mice immunized with *H. suis* whole-cell lysate and protected against infection were identified by using two-dimensional (2D) gel electrophoresis followed by immunoblotting and LC-MS/MS. Sera of *H. suis*- infected mice were also included, since an infection does not result in protection. Based on this analysis, the immunoreactive *H. suis* urease subunit B (UreB) was selected for further in vivo testing. As a control we included the *H. suis* neutrophil-activating protein A (NapA), which has been previously described as a possible virulence factor [9] but was not recognized by sera of mice immunized with whole-cell lysate. Subsequently, the protective efficacy against an *H. suis* infection of both subunit vaccines was evaluated and compared with that of *H. suis* lysate in a standardized mouse model.

## Materials and methods

### Bacterial strain

In all experiments, *H. suis* strain 5 (HS5, GenBank: ADHO00000000) was used. This strain was isolated from the gastric mucosa of a pig according to the method described by Baele et al. [10].

### Animals

One week prior to the initiation of the experiments, five-week-old specific-pathogen-free female BALB/c mice were obtained from an authorized breeder (HARLAN, Horst, The Netherlands). The animals were housed on sterilized wood shavings in filter top cages. They were fed with an autoclaved commercial diet (TEKLAD 2018S, HARLAN) and received autoclaved water *ad libitum*. All laboratory animal experiments were approved by the Animal Care and Ethics Committee of the Faculty of Veterinary Medicine, Ghent University.

### Immunoproteomics of *H. suis*

#### Two-dimensional gel electrophoresis (2D-PAGE)

HS5 was grown as described previously [11]. Bacteria were harvested by centrifugation (5000 *g*, 4°C for 10 min) and washed four times with Hank’s balanced salt solution (HBSS). Total proteins (both soluble and insoluble proteins) were extracted in two steps using the ReadyPrep™ Sequential Extraction Kit (Bio-Rad, Hercules, CA, USA) according to manufacturer’s instructions. In order to obtain good 2D-PAGE results, the homogenates were treated with proper additives (5 mg protease inhibitor cocktail, 1 μL DNAse I, 1 μL RNAse A, 10 μL phosphatase inhibitors PP2 and PP3 (Sigma-Aldrich, Steinheim, Germany)). Finally, the protein concentration was determined using the *RC DC* Protein Assay (Bio-Rad) and proteins were stored at −70°C till further use. A total of 100 μg of HS5 proteins were rehydrated in 200 μL rehydration buffer (7M ureum, 2M thioureum, 2% CHAPS, 0.2% carrier ampholyte pH3-4, 100mM dithiothreitol (DTT) and bromophenol blue). Samples were passively absorbed into a ReadyStrip (11 cm, pH3 to pH10, Bio-Rad) and iso-electric focusing was carried out in a Protean IEF Chamber (Bio-Rad) as previously described [12]. After iso-electric focusing, the strips were equilibrated for 15 min in 1.5% DTT in equilibration buffer (50mM TrisHCl, pH 8.8 6M urea, 20% glycerol, 2% SDS) followed by another equilibration in 4% iodoacetamide in equilibration buffer. Gel electrophoresis was carried out on a 10% TrisHCl SDS-PAGE using 150V for 30 min, followed by 200V for 1 h. Two gels were run in parallel: one was stained with Sypro® Ruby Protein Gel staining (Bio-Rad) while the other was used for immunoblotting (see Western blotting described below). Prior to staining, gels were fixed in 10% MeOH, 7% acetic acid. After staining, *H. suis* proteins were visualized using the VersaDoc Imaging System (Bio-Rad).

#### Serum pools

Three pools of mouse sera were used in this study:

Sera from mice immunized with *H. suis* whole-cell lysate (hereafter referred to as “lysate-immunized mice”) (*n* = 10). These animals were inoculated intranasally twice with three weeks interval with 100 μg HS5 lysate + 5 μg cholera toxin (CT) (List Biological Laboratories Inc., Madison, NJ, USA). HS5 lysate was prepared as described previously but without final filtration of the supernatant [6]. Three weeks after the last immunization, blood was collected and sera were pooled. This immunization protocol has been shown to be (partially) protective against *H. suis* challenge [6] and the protective effect was confirmed here in a preliminary experiment (data not shown).

Sera from *H. suis*-infected mice (hereafter referred to as “infected mice”) (*n* = 10). These animals were inoculated intragastrically with 200 μL Brucella broth at pH 5, containing 10^8^ freshly prepared *H. suis* bacteria [11]. Four weeks after infection, blood was collected and sera were pooled.

Sera from negative control mice (*n* = 10). These animals received HBSS intranasally twice with a three weeks interval followed by intragastric inoculation with 200 μL Brucella broth at pH5 (4 weeks after last sham immunization). After four weeks, blood was collected and sera were pooled.

All sera were stored at −70°C until further use.

#### Western blotting

Proteins were electrotransferred from gels onto nitrocellulose membranes (Bio-Rad) as described elsewhere [12]. Membranes were blocked in 5% skimmed milk in phosphate buffered saline (PBS) (blocking buffer), incubated overnight (ON) with diluted mouse sera (1/100 in blocking buffer) at room temperature (RT), rinsed in PBS with 0.3% Tween-20 (wash buffer) and incubated for 1 h at RT with stabilized goat anti-mouse immunoglobulin G (IgG) horseradish-peroxidase (HRP)-conjugated (1/1000 in blocking buffer, Pierce, Rockford, IL, USA). After a wash step in wash buffer, immunodetection of proteins was performed by enhanced chemiluminescence detection using Supersignal West Dura Extended Duration Substrate (Pierce). Protein patterns were scanned and digitized using the VersaDoc Imaging System. All experiments were performed in triplicate.

#### In-gel protein digestion and identification by mass spectrometry

In-gel digestion of proteins was performed as described by Cheung et al. [13]. Prior to mass spectrometry the isolated peptides were separated on a U3000 nano-high-performance liquid chromatography (HPLC) (Dionex, Sunnyvale, CA, USA) as previously described [14].

Identification of the peptides was performed using an electrospray ionization quadrupole time-of-flight mass spectrometry (ESI-Q-TOF) Ultima (Waters, Milford, MA, USA) as described previously [14]. Data analysis was performed against the *Helicobacter* protein database from NCBI (146 612 entries) using the in-house search engine Mascot Daemon (2.3, Matrix Science, London, UK). An error tolerant search was performed with carbamidomethyl (C) as fixed modification. Carbamidomethyl (N-terminal) and oxidation (M) were set as variable modifications. Peptide mass tolerance and fragment mass tolerance was set at 0.35 Da and 0.45 Da, respectively. Maximum two miscleavages were allowed. Proteins were only considered to be correctly annotated when the significance was below 0.05 (*p* < 0.05) and at least one peptide passed the required bold red criteria from Mascot Daemon, indicating that at least one peptide had rank 1 and a significance below 0.05.

#### One-dimensional gel electrophoresis (1D-PAGE) and Western blotting of rUreB

1D-PAGE of 10 μg recombinant *H. suis* urease subunit B (rUreB) was performed as described by Van Steendam et al. [12]. Sera preparation and Western blot analyses were performed as described above.

### Protective efficacy of recombinant *H. suis* proteins in a mouse model

#### Preparation of recombinant UreB

A fragment encoding the *H. suis* UreB sequence (GenBank locus tag HSUHS5_0285) was amplified by PCR using a Pwo polymerase with proofreading activity (Roche, Mannheim, Germany) from the DNA of HS5 (forward primer: 5’- ATG AAA AAA ATC TCT AGG AAA GAA TAT G -3’; reverse primer: 5’- CTA GTG ATG GTG ATG GTG ATG GAA CAA GTT GTA GAG TTG AGC -3’) and cloned into the protein expression vector pET-24d. The rUreB was expressed in *E. coli* strain BL21 (DE3). The cells were lyzed by sonication (5 times for 30 s) in buffer containing 50mM Na.PO_4_ pH7, 0.5M NaCl, 1M DTT, 1% Triton X-100 and 1mM PMSF. After centrifugation (4°C, 20 000 *g* for 30 min), rUreB was purified from the soluble fraction using Ni-affinity chromatography in buffer consisting of 1M NaCl, 50mM PBS, 1% Triton X-100, 250mM imidazole and 10% glycerol (His GraviTrap, GE Healthcare Bio-Sciences AB, Uppsala, Sweden) followed by gel filtration on a Superdex™ 200 HR 16/60 column (GE Healthcare Bio-Sciences AB). After purification, rUreB was analyzed using SDS-PAGE and Western blot analysis using anti-hexahistidine-tag mouse monoclonal antibody (Icosagen Cell Factory, Tartu, Estonia). The detergent Triton X-100 was removed from the purified rUreB by using Pierce Detergent Removal Spin columns (Pierce) following manufacturer’s instructions. Protein concentration was determined with the *RC DC* protein Assay (Bio-Rad).

#### Preparation of recombinant NapA

The protein was expressed in the *E. coli* Expression System with Gateway® Technology (Invitrogen, Carlsbad, CA, USA) as follows. A fragment encoding the *H. suis* neutrophil-activating protein A (NapA) sequence (GenBank locus tag HSUHS5_0014) was amplified by PCR using a Pwo polymerase with proofreading activity (Roche) from the DNA of HS5 (forward primer: 5’- CACCATG AAAGCAAAAACAGTTGATGTACTC -3’; reverse primer: 5’- TTAAGCCAAACTTGCCTTAAGCATCC -3’) and cloned into the pENTR™/TEV/D-TOPO® vector and transferred into the pDEST17™ destination vector. The selected pDEST17-NapA plasmid was transformed to the BL21-AI™ *E. coli* and subsequently grown at 37°C to an OD_600_ of 0.6-1.0 in Luria Broth supplemented with 50 μg/mL carbenicillin. Recombinant *H. suis* NapA (rNapA) expression was induced by adding 0.2% L- arabinose. After 4 h incubation at 37°C, the cells were harvested and resuspended in lysis buffer: 50mM TrisHCl, 100mM NaCl, 1% Triton X-100, 0.2 mg/mL lysozyme, 20 μg/mL DNAse, 1mM protease inhibitor (Sigma), and 1mM MgCl_2_. The cells were lyzed by sonication (5 times for 30 s). Cell debris and inclusion bodies were isolated by centrifugation at 4°C (20 000 *g* for 30 min). The inclusion bodies were subsequently washed twice based on the following protocol: the pellet was resuspended in cold lysis buffer, sonicated 5 times for 30 s followed by centrifugation (4°C, 20 000 *g* for 30 min). The washed inclusion bodies were solubilized in binding buffer, pH8 (6M guanidium HCl, 20mM TrisHCl, 0.5M NaCl, 5mM imidazole, 1mM β-mercaptomethanol) by gentle rotation for 1 h at RT. Insoluble material was removed by high speed centrifugation at 4°C (100 000 *g* for 30 min). rNapA was purified from the clarified supernatant onto a Ni-sepharose column (His GraviTrap, GE Healthcare Bio-Sciences AB) according to the manufacturer’s instructions. rNapA was eluted with elution buffer, pH8 (8M urea, 20mM TrisHCl, 0.5M NaCl, 0.5M imidazole, and 1mM β-mercaptoethanol) and ON dialyzed against PBS at 4°C. Afterwards, rNapA was analyzed using SDS-PAGE and protein concentration was determined using *RC DC* Protein Assay (Bio-Rad).

#### Immunization and infection experiments

**Figure 1:**
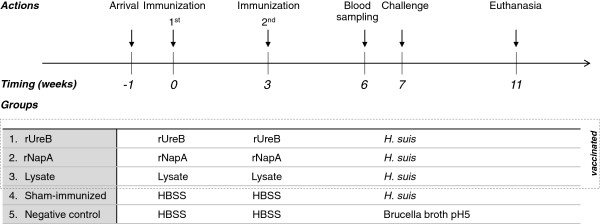
**Experimental design of vaccination study.** Per group 10 mice were intranasally immunized twice with 3 weeks interval, each time with 30 μg rUreB + 5 μg cholera toxin (CT); 30 μg rNapA + 5 μg CT, and 100 μg HS5 lysate + 5 μg CT (groups 1, 2 and 3, respectively). Groups 4 (sham-immunized group) and 5 (negative control group) were intranasally inoculated with HBSS. Three weeks after the second immunization, blood was collected from 5 mice per group and one week later mice of groups 1, 2, 3 and 4 were intragastrically inoculated with 10^8^ viable *H. suis* bacteria. Group 5 was intragastrically inoculated with HBSS. Four weeks after intragastric challenge, mice were euthanized.

The experimental design is summarized in Figure 1. Five groups of 10 mice were intranasally inoculated twice with 3 weeks interval, each time with 17.5 μL inoculum. In groups 1, 2 and 3 the inoculum consisted of HBSS with 5 μg CT, containing 30 μg rUreB, 30 μg rNapA and 100 μg HS5 lysate, respectively. Groups 4 (sham-immunized group) and 5 (negative control group) were inoculated with HBSS. Three weeks after the second intranasal immunization, blood was collected by tail bleeding from five animals per group and one week later, all animals, except the negative control group, were inoculated intragastrically with 200 μL Brucella broth at pH 5 containing 10^8^ viable *H. suis* bacteria [11]. The negative control group was inoculated intragastrically with 200 μL Brucella broth at pH5. Four weeks after the intragastric inoculation, mice were euthanized by cervical dislocation following isoflurane anaesthesia (IsoFlo; Abbott, IL, USA). From the euthanized animals, blood was collected by sterile cardiac puncture, centrifuged (1000 *g*, 4°C, 10 min) and serum was frozen at −70°C until further use. Stomachs were excised and dissected along the greater curvature. One-half of the stomachs, including antrum and fundus, was immediately placed into 1 mL RNA Later (Ambion, Austin, TE, USA) and stored at −70°C for further RNA- and DNA-extraction. A longitudinal strip of the gastric tissue was cut from the oesophagus to the duodenum along the greater curvature for histopathological examination.

#### Quantification of *H. suis* in the stomach

After thawing, stomach tissues were homogenized (MagNAlyser, Roche, Mannheim, Germany) in 1 mL Tri Reagent® RT (MRC, Brunschwig Chemie, Amsterdam, The Netherlands) and DNA was extracted from the inter- and organic phase according to Tri Reagent® RT manufacturer’s instructions. The bacterial load in the stomach was determined using the previously described *H. suis* specific quantitative real-time PCR (qPCR) [5].

#### Analysis of stomach cytokine response

The expression levels of IFN-γ, IL-4, IL-10, IL-17 and TNF-α were assessed by qPCR using cDNA synthesized from stomach tissue as described previously [15]. The threshold cycle (Ct) values were normalized to the geometric mean of the Ct-values from the reference genes after which normalized mRNA levels were calculated using the 2^-ΔΔCt^ method [16].

#### Measurement of serum antibody responses by enzyme-linked immunosorbent assay (ELISA)

The Protein Detector™ ELISA Kit (KPL, Gaithersburg, MD, USA) was used to evaluate rUreB-, rNapA-, and HS5 lysate specific IgG in serum. In brief, 96 well flat bottom plates (Nunc MaxiSorp, Nalge Nunc Int., Rochester, NY, USA) were coated with 2 μg/well of purified rNapA, 1 μg/well of purified rUreB, or 1 μg/well of *H. suis* whole cell proteins diluted in 100 μL coating buffer (24 h, 4°C). After blocking with 1% bovine serum albumin in PBS, 100 μL of 1/400 diluted serum was added to each well. After further washing, 100 μL of HRP-labeled anti-mouse IgG (H+L) in a final concentration of 50 ng per well was added. Five minutes after adding 2,2'-azino-bis(3-ethylbenzothiazoline-6-sulphonic acid) (ABTS) peroxidase substrate solution, absorbance was read at 405nm (OD_405nm_).

#### Histopathological examination

The longitudinal gastric tissue strips were fixed in 4% phosphate buffered formaldehyde, processed by standard procedures and embedded in paraffin. For evaluation of gastritis, haematoxylin - eosin (HE) stained sections of 5 μm were blindly scored based on the degree of infiltrating lymphocytes, plasma cells and neutrophils, using a visual analog scale similar to the Updated Sydney System (on a scale of 0–3) [17] with the following specifications for each gastritis score: 0 = no infiltration of mononuclear and/or polymorphonuclear cells; 1 = mild diffuse infiltration of mononuclear and/or polymorphonuclear cells; 2 = moderate diffuse infiltration of mononuclear and/or polymorphonuclear cells and/or the presence of one or two inflammatory aggregates; 3 = marked diffuse infiltration of mononuclear and/or polymorphonuclear cells and/or the presence of at least three inflammatory aggregates.

#### Statistical analysis

Normality and variance homogeneity of data was analyzed by using D’Agostino-Pearson and Shapiro-Wilk normality test. Significant differences in *H. suis* colonization and mRNA cytokine expression among groups were assessed by performing one-way ANOVA analysis. Bonferroni’s multiple comparison test was used as post-hoc when equal variances were assessed. Dunnett’s T3 post-hoc test was used when no equal variances were assessed. OD_405nm_ levels from ELISA and histological inflammation scores were compared by Kruskall-Wallis analysis, followed by a Mann–Whitney *U* test. For correlations between different variables, Spearman’s rho coefficient (*ρ*) was calculated. GraphPad Prism5 software (GraphPad Software Inc., San Diego, CA, USA) was used for all analyses. Statistically significant differences between groups were considered at *p* < 0.05.

## Results

### Immunoproteomics of *H. suis*

**Figure 2:**
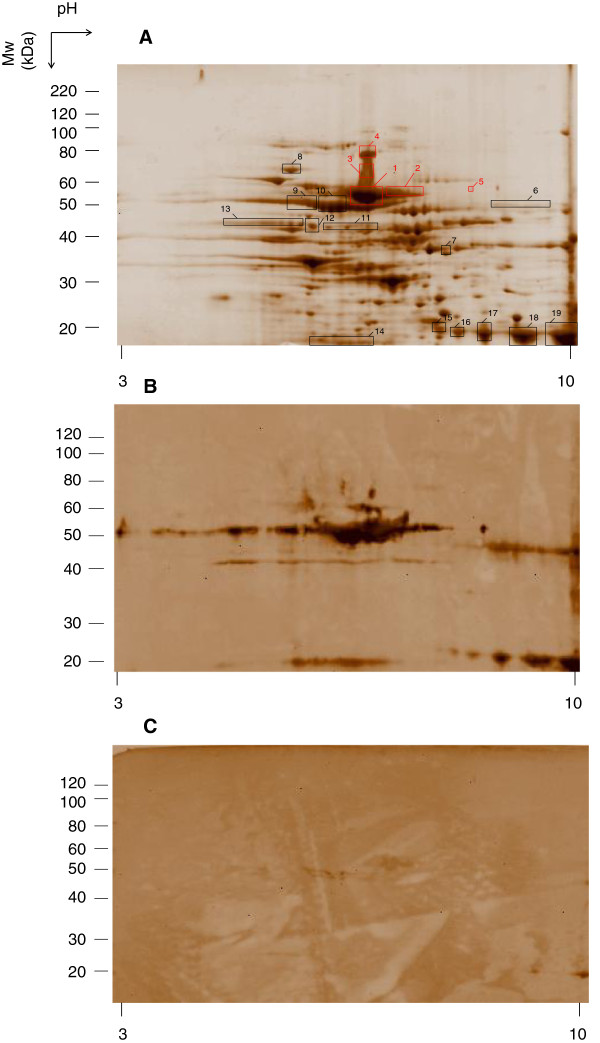
***H. suis *2D-proteome profile (A) and Western blots of a duplicate 2D-gel reacted with pooled sera of lysate-immunized mice (B) or of *H. suis*-infected mice (C).** 100 μg of total protein extract of *H. suis* was separated by 2D-electrophoresis using linear pH3 to10 gradient in the first dimension and 10% TrisHCl SDS-PAGE in the second dimension. The separated proteins were detected by SYPRO®Ruby Protein staining. The boxed areas indicate where immunoreactive antigens were excised from the gel and subjected to LC-MS/MS. Identified proteins are indicated by the spot numbers given in Table 1. Boxes and numbers in red were identified as UreB. The position of molecular weight (MW) is given on the right, and the pH is given at the bottom.

*H. suis* proteins were separated on 2D-PAGE (Figure 2a). After 2D-immunoblotting with pooled sera from lysate-immunized (Figure 2b) or *H. suis*-infected animals (Figure 2c), a total of 19 immunoreactive protein spots were selected. These spots were matched with the protein spots that could be seen in the parallel 2D-PAGE (Figure 2a). Little reactivity against *H. suis* proteins was observed in post-infection sera compared to the high reactivity against sera from lysate-immunized mice. When the blot was probed with a pool of sera obtained from negative control mice, no specific immunoreactive protein spots were detected (Additional file 1). Spots of interest (*n* = 19) were cut out of the gel, digested and identified by means of LC-MS/MS analysis. The detailed results of these proteins are summarized in Table 1. Spots with the highest reactivity (spot 1 to 5) were identified as UreB. *H. suis* chaperonin GroEL, illustrated as spots 9 and 10 on Figure 2a, showed also strong hybridization with sera from lysate-immunized animals. Additionally, sera from lysate-immunized mice showed strong reactivity against the urease accessory protein (UreH) and the urease subunit A (UreA) (spots 15 to 19), which was less pronounced in the infected group. Weak reactivity against the major flagellin FlaA (spots 11 to 13) was present in both blots.

**Table 1:** **Immunoreactive proteins of *H. suis *identified by LC**-**MS**/**MS**

**Spot no**.^**1**^	**Protein name**	**NCBI ID**^**2**^	**Gene**	**pI**^**3**^	**MW**^**3**^	**No. matched peptides**	**Mascot score**^**4**^	**Cov. (%)**^**5**^
**1**	Urease subunit B	EFX42254	*ureB*	5.97	62.967	117	1589	72
**2**	Urease subunit B	EFX42254	*ureB*	5.97	62.967	80	1042	58
	Threonyl-tRNA synthetase	EFX41598	*thrS*	6.34	69.315	7	186	60
**3**	Urease subunit B	EFX42254	*ureB*	5.97	62.967	80	903	46
**4**	Urease subunit B	EFX42254	*ureB*	5.97	62.967	4	103	6
**5**	Urease subunit B	EFX42254	*ureB*	5.97	62.967	4	103	6
**6**	30S ribosomal protein S1	EFX42427	*rpsA*	8.29	64.051	23	625	28
	Quinone-reactive Ni/Fe hydrogenase, large subunit	EFX41851	*hydB*	8.22	64.943	19	520	28
	Urease of *H. heilmannii*	AAA65722		8.86	25.844	8	258	26
**7**	Methyl-accepting chemotaxis protein	EFX43528		7.1	48.907	35	905	50
**8**	Elongation factor G	EFX41637	*fusA*	5.15	77.242	57	1066	55
**9**	Chaperonin GroEL	EFX42237	*groEL*	5.58	58.498	150	3085	78
**10**	Chaperonin GroEL	EFX42237	*groEL*	5.58	58.498	135	2647	73
	Urease subunit B	EFX42254	*ureB*	5.97	62.967	43	682	41
**11**	Flagellin A	EFX41982	*flaA*	7.77	54.232	29	756	47
**12**	Flagellin A	EFX41982	*flaA*	7.77	54.232	57	1294	62
**13**	Flagellin A	EFX41982	*flaA*	7.77	54.232	46	1085	63
	Trigger factor	EFX42378	*tig*	5.13	49.587	12	343	24
**14**	Hydrogenase expression/formation protein	EFX41790	*hypB*	5.59	27.617	15	314	35
	Nicotinate-nucleotide pyrophosphorylase	EFX42191	*nadC*	6.46	30.591	11	219	25
	7-alpha-hydroxysteroid dehydrogenase	EFX41880		6.62	28.246	6	186	24
	Conserved hypothetical secreted protein	EFX42511	*hdhA*	5.84	28.011	5	174	19
	Hypothetical protein HSUHS5_0308	EFX42276		5.47	29.471	9	171	24
	Peroxiredoxin	EFX42277		5.84	25.811	7	107	19
**15**	Urease accessory protein	EFX42255	*ureH*	6.79	30.447	4	106	13
**16**	Urease subunit A	EFX42255	*ureA*	7.79	27.389	24	270	55
**17**	Urease subunit A	EFX42255	*ureA*	7.79	27.389	28	112	45
**18**	Urease subunit A	EFX42255	*ureA*	7.79	27.389	57	418	69
**19**	Urease subunit A	EFX42255	*ureA*	7.79	27.389	75	568	73

^1^ Protein spot corresponding to position on gel and blots (see Figure 2).

^2^*NCBI*: National Center for Biotechnology Information.

^3^ Theoretical isoelectric point (pI) and molecular weight (MW).

^4^ For *Helicobacter* data, Mascot scores greater than 40 are significant (*p* ≤ 0.05).

^5^ % of the protein sequence covered by the peptides identified.

### Confirmation of serum reactivity against rUreB

From the 2D-analysis, UreB showed distinct reactivity with sera from lysate-immunized mice, which was not observed in sera from non-immunized but infected mice. In order to confirm these data, a 1D-PAGE loaded with rUreB was performed, followed by immunodetection with sera from lysate-immunized and *H. suis*-infected mice. Reactivity was only detected in the immunized group and a distinct band was visible at ~ 63 kDa, which corresponds to the molecular weight of UreB (Additional file 2).

### Protective efficacy of recombinant *H. suis* proteins in a mouse model

From the 2D-proteomics approach, *H. suis* UreB was found to show a high reactivity with sera from lysate-immunized mice. Therefore, this protein was selected for further in vivo analyses. In addition, the *H. suis* NapA was tested. NapA has been previously described as a possible virulence factor of *H. suis*[9], but was not detected by sera of lysate-immunized mice.

**Figure 3:**
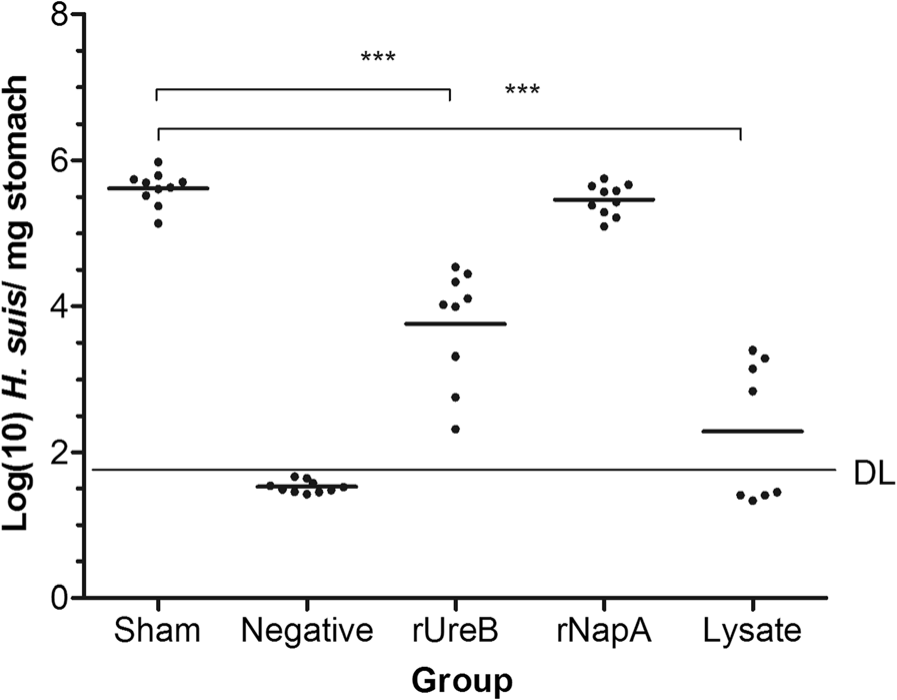
**Protection against *H. suis *challenge after prophylactic intranasal immunization.** Bacterial load is illustrated as log (10) of *H. suis* copies/mg stomach tissue. Individual mice are illustrated as dots around the mean (lines). DL: detection limit of 43.9 copies mg^-1^. Significant differences between immunized (rUreB, rNapA and lysate) and sham-immunized, infected animals are noted by *** *p* < 0.001. Results of negative controls were all situated below DL.

During the immunization experiment but before intragastric challenge, one animal from the rUreB immunized group and two animals from the group immunized with lysate died. The protective efficacy of rUreB, rNapA and lysate is shown in Figure 3 and expressed as the number of *H. suis* copies detected by qPCR in the stomach of challenged mice. High levels of *H. suis* (> 10^5^ copies mg^-1^ stomach) were detected in the stomach of sham-immunized mice. Prophylactic immunization with rUreB induced a significant reduction in *H. suis* colonization compared to sham-immunized mice (*p* < 0.001). In contrast, immunization with rNapA did not induce a significant reduction (*p* = 0.14) in bacterial colonization. Immunization with lysate resulted in a significant reduction of the bacterial load (*p* < 0.001), and in 50% of the animals *H. suis* DNA was not detected by qPCR. A significant lower gastric bacterial load was observed in lysate-immunized mice compared to rUreB- and rNapA-immunized mice (*p* < 0.01).

### Stomach cytokine response

**Figure 4:**
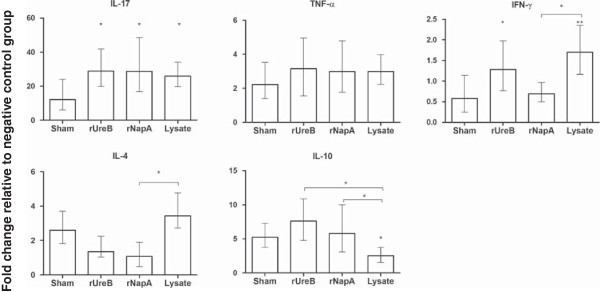
**Fold change in cytokine gene expression level in the stomach relative to negative control animals.** The stomach mRNA expression levels of cytokines (IL-4, IL-10, IL-17, IFN-γ and TNF-α) at final euthanasia were examined by qPCR. Data represent the normalized target gene amount relative to the negative control group which is considered 1. Data are shown as means ± standard error of mean. Significant differences between immunized (rUreB, rNapA and lysate) and sham-immunized, infected animals are noted by * *p* < 0.05 and ** *p* < 0.01. Significant differences between immunized groups are noted by bars and * *p* < 0.05.

mRNA expression levels of cytokines (IFN-γ, TNF-α, IL-4, IL-10, IL-17) in gastric tissue are illustrated in Figure 4. Expression of IL-17, a marker for a Th17 response, was increased (*p* < 0.05) in all immunized groups compared to sham-immunized mice. The IFN-γ response was significantly higher in rUreB- and lysate- immunized groups compared to the sham-immunized group (*p* < 0.05 and *p* < 0.01 respectively). Immunization with rNapA did not result in increased IFN-γ expression levels compared to sham-immunization (*p* > 0.05). When taking all groups inoculated intragastrically with *H. suis* into account (rNapA, rUreB, lysate and sham), a significant inverse correlation was observed between IL-17 and IFN-γ response on the one hand, and colonization on the other hand (*ρ* = −0.388 and *ρ* = −0.816, respectively, *p* < 0.05). IL-10 expression levels in the lysate-immunized group were significantly lower (*p* < 0.05) compared to all other groups (rUreB, rNapA and sham). A significant correlation was observed between IL-10 response and gastric colonization (*p* < 0.01, *ρ* = 0.427). IL-4 expression levels were higher in sham- and lysate-immunized groups compared to rNapA- and rUreB- immunized groups. This was significantly (*p* < 0.05) higher in lysate-immunized mice compared to rNapA-immunized mice. For TNF-α no significant differences in expression were observed between immunized and sham-immunized groups.

### Specific serum antibody response before and after challenge

**Figure 5:**
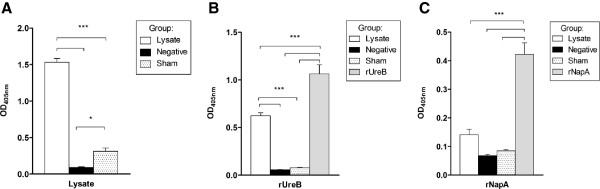
**Serum antibody responses against lysate (A), rUreB (B) or rNapA (C) at euthanasia.** The levels of specific IgG are shown as the mean OD_405 nm_ + SD. * *p* < 0.05, *** *p* < 0.001.

Three weeks after the last immunization and at euthanasia, serum was prepared for analysis of the serum-IgG response against rNapA, rUreB, and lysate. Serum levels of anti- rNapA, - rUreB and - lysate IgG of mice immunized with respective antigens were significantly elevated compared to negative controls at 3 week post-immunization (Additional file 3) and to both negative controls and sham-immunized mice at final euthanasia (Figure 5). Mice immunized with lysate showed a rUreB-specific serum IgG response but no rNapA-specific response (Figure 5b and c). When taking all immunized groups into account (rNapA, rUreB and lysate), a significant inverse correlation (*ρ* = −0.783, *p* < 0.001) between specific IgG and *H. suis* copies mg^-1^ stomach was observed.

### Histopathology

The overall gastric inflammation scores are presented in Table 2. All negative control mice showed normal histomorphology with very little inflammatory cell infiltration in the gastric mucosa. Sham-immunized mice developed a weak to moderate gastric inflammation. In general, higher inflammation scores were observed in the fundus compared to the antrum. Mice immunized with lysate showed a weak to moderate gastric inflammation, which was not significantly different from that observed in sham-immunized infected mice (*p* > 0.3). Although not significant (*p* > 0.05), less severe inflammatory infiltration was observed in rNapA and rUreB-immunized mice compared to mice immunized with lysate and sham-immunized mice.

**Table 2:** Gastric inflammation scores in mice after immunization and infection

**Group**	**Inflammation score fundus**^**1**^				**Inflammation score antrum**^**1**^				**Overall mean inflammation score/group**
	**0**	**1**	**2**	**3**	**0**	**1**	**2**	**3**	
**rUreB**	1	6	2	0	7	2	0	0	0.66
**rNapA**	4	6	0	0	8	2	0	0	0.40
**Lysate**	1	4	3	0	2	5	1	0	1.06
**Sham-immunized**	1	5	4	0	5	5	0	0	0.90
**Negative control**	10	0	0	0	10	0	0	0	0.00

^1^Shown are the number of animals per group with a specific overall inflammation score in antrum and/or fundus: animals vaccinated with rUreB (*n* = 9), rNapA (*n* = 10) or lysate (*n* = 8), sham-immunized and infected controls (*n* = 10) and negative controls (*n* = 10). 0, no infiltration of mononuclear and/or polymorphonuclear cells; 1, mild diffuse infiltration of mononuclear and/or polymorphonuclear cells; 2, moderate diffuse infiltration of mononuclear and/or polymorphonuclear cells and/or the presence of one or two inflammatory aggregates; 3, marked diffuse infiltration of mononuclear and/or polymorphonuclear cells and/or the presence of at least three inflammatory aggregates.

## Discussion

We previously demonstrated that immunization with *H. suis* whole-cell lysate protected mice against a subsequent experimental *H. suis* infection and resulted in high serum anti-*H. suis* IgG titers [6]. An *H. suis* infection, on the other hand, did not result in protective immunity, whereby significantly lower serum IgG titers were observed compared to *H. suis* protected animals [6]. In order to identify possible vaccine candidates, 2D-gel electrophoresis of *H. suis* proteins was performed followed by immunoblotting with pooled sera from *H. suis*- infected mice or mice immunized with *H. suis* whole-cell lysate. To our knowledge, this is the first study describing the immunoproteome of *H. suis*. The UreB protein showed a pronounced reactivity against sera from immunized mice and was not detected with sera from infected mice (Figure 2b and c). This protein was therefore selected for further evaluation of its protective efficacy. We found that immunization with rUreB resulted in a significant reduction of *H. suis* colonization in the stomach. The urease protein is known to be crucial for the survival of gastric *Helicobacter* species [2,18] and vaccination with its subunit B (either natural or recombinant) also induced partial protection against *H. pylori*, *H. felis* and *H. heilmannii*[19-23].

Vaccination with rUreB did not induce complete protection against an experimental *H. suis* infection. In contrast, a complete clearance was observed in 50% of mice immunized with whole-cell lysate, which is in line with previously observed results [6]. Most probably, in order to obtain a degree of protection which is similar to or better than that induced by whole-cell lysate, additional *H. suis* antigens will have to be included in subunit vaccines. The *H. suis* chaperonin GroEL (spots 9 and 10) is another protein that showed strong reactivity with sera from lysate-immunized mice and might therefore also be a candidate for inclusion in a subunit vaccine. Indeed, oral vaccination with *H. pylori* Hsp60 or *E. coli* GroEL induced a partial protection against *H. pylori* challenge [24,25]. However, vaccination with this protein has also been associated with post-immunization gastritis [25]. Additionally, it has been demonstrated that antibodies against *H. pylori* Hsp60 may be associated with gastric cancer and - inflammation in humans [26-28]. Other immunoreactive protein spots identified in this study include UreA, UreH, FlaA, trigger factor, hydrogenase expression/formation protein, methyl-accepting chemotaxis protein and elongation factor G. All these proteins have also been identified in immunoproteomic studies of *H. pylori* and seem to be essential for gastric colonization of this bacterium [29]. Future research is needed to determine the protective efficacy and possible side effects of vaccination with (combinations of) these proteins.

NapA has been recognized as a key modulator in *H. pylori*-induced gastritis [30] and has been proposed as a protective antigen and promising vaccine candidate against *H. pylori* infections [31,32]. In the present study, NapA was not recognized by the pooled sera from lysate-immunized mice and intranasal immunization with rNapA did not result in protection against *H. suis* challenge, although it induced anti-rNapA IgG. The reason for the different outcome in protection studies with this protein in *H. suis* and *H. pylori* remains unclear. Differences in vaccine preparations, adjuvants and experimental infection models used may play a role. Although the *H. suis napA* gene shows strong homology with its *H. pylori* equivalent (99% of sequence aligned, of which 83% conserved) [9], the role of NapA in the pathogenesis of *H. suis* infections has not yet been determined and is not necessarily identical to that of *H. pylori*.

Different immune mechanisms may be involved in protection induced by the vaccines tested here. Serum antibodies against rUreB or antigens present in *H. suis* lysate were detected in mice vaccinated with rUreB or lysate, respectively, while they were absent (rUreB) or remarkably lower in non-vaccinated, infected mice. In future studies it may be interesting to also determine IgA antibody titers locally produced in the stomach. The role of local and serum antibodies in protection against a *Helicobacter* infection is, however, controversial. Although several authors mentioned that they may play a role in protection [33-38], results of other studies indicate that prophylactic immunization against *Helicobacter* species does not require antibodies [39,40]. Whether circulating and/or local antibodies play a role in protection against *H. suis* infections may be determined by using antibody-deficient mice or by passive administration of serum antibodies [34,35,39-41].

In mice vaccinated with rUreB or lysate, mRNA expression of IFN-γ, a signature Th1 cytokine, was significantly higher after challenge with *H. suis* compared to sham-immunized mice, and this was not demonstrated for rNapA-immunized, not protected mice. Moreover, a clear inverse correlation was observed between the bacterial load and IFN-γ mRNA expression levels. In non-vaccinated mice, an *H. suis* infection does not induce a Th1 response and does not result in clearance of the infection [15]. This indicates that production of IFN-γ, elicited by immunization, could play a role in suppression and clearance of *H. suis.*

Expression levels of IL-17 after challenge with *H. suis* were elevated in mice immunized with rUreB, rNapA and lysate, compared to sham-immunized mice and also for this cytokine, an inverse correlation with *H. suis* colonization was observed. In non-vaccinated mice, an *H. suis* infection mainly results in a Th17 response and a secondary Th2 response, which is not able to eradicate the infection although the Th17 response inversely correlates with bacterial load [15]. This might indicate that for a strong suppression or clearance of *H. suis*, a combined Th17 and Th1 response in the stomach is necessary, as was observed in the rUreB- and lysate-vaccinated groups.

We observed that decreased expression levels of IL-10 were correlated with a reduction in gastric *H. suis* colonization. This is not entirely unexpected, since IL-10 is a suppressive cytokine for Th17 and Th1. Additionally, it has been shown that IL-10-deficient mice are able to eradicate *H. pylori* infection [42].

After infection with *H. suis*, expression of IL-4, a marker of a Th2 response, was higher in the lysate-immunized group than in groups vaccinated with rUreB and rNapA. Taken all results of the present study together, there are indications that in addition to a local Th1 and Th17 response, a Th2 response, probably resulting in local production of antibodies, may help to eradicate *H. suis* from the stomach. Indeed, only in the lysate-immunized group, mice were able to clear *H. suis* from the stomach. Further studies are, however, necessary to confirm this hypothesis.

In lysate-immunized mice, *H. suis* colonization was significantly lower than in the other experimentally infected groups. However, histological examination revealed that the inflammatory response in this group was almost similar to that in sham-immunized, *H. suis*-infected mice. For *H. pylori* too, a transient gastritis is often seen after challenge of immunized mice [43]. It remains to be investigated whether gastritis levels of lysate-immunized mice would drop below gastritis levels of sham-immunized animals after a longer period post challenge.

In conclusion, sera from lysate-immunized, protected mice strongly react with *H. suis* UreB and immunization with this antigen induced a significant reduction in gastric *H. suis* colonization in challenged mice. Although rUreB is a promising antigen candidate for the use in vaccines against *H. suis* infections, further studies are necessary to elucidate if inclusion of additional *H. suis* antigens may improve the protective efficacy of subunit vaccines. Also, results obtained in this mouse model should be confirmed in pigs, which are the natural host of *H. suis*. Probably, a combination of local Th1 and Th17 responses, complemented by antibody responses play a role in the protective immunity against *H. suis* infections. The exact mechanism by which protection against an *H. suis* infection is mediated remains however to be elucidated.

## Competing interests

The authors declare that they have no competing interests.

## Authors’ contributions

MV participated in the design of the study, performed in vivo and in vitro experiments, analyzed data and drafted the manuscript. KVS performed LC-MS/MS analyses and coordinated the immunoproteomics experiments. BF participated in the design, coordination and analyses of the study. AS participated in the design of the study. PG contributed with the coordination of immunoproteomics experiments. FP, RD and DD coordinated and participated in the design of the study. FH coordinated and participated in the design of the study, helped to interpret the results and edited the manuscript. All authors read and approved the final manuscript.

## Authors’ information

Dieter Deforce and Freddy Haesebrouck shared senior authorship.

## Supplementary Material

Additional file 1 **Immunodetection of a 2D-Western blot with a pool of control sera from *H. suis *-negative mice.** 100 μg of *H. suis* total protein extract was separated by 2D-electrophoresis using linear pH3 to10 gradient in the first dimension and 10% SDS-PAGE in the second dimension. After transfer of the proteins onto a nitrocellulose membrane, the 2D-immunoblot was analyzed by reacting with a pool of control sera from 10 *H. suis*-negative mice. No specific immunoreactive protein spots were detected. Click here for file

Additional file 2 **1D-PAGE immunoblotting of rUreB.** M: Protein marker. Lane 1 and 2: 10 μg rUreB separated on 10% TrisHCl SDS-PAGE and immunoblotted with serum of mice 3 weeks after immunization with *H. suis* whole-cell lysate (1) or with serum of *H. suis*-infected mice at four weeks post-infection (2). Both sera consisted of a pool of 10 animals. Only in serum of immunized animals (lane 1) immunoreactivity against rUreB is seen as a ~ 63 kDa band. Click here for file

Additional file 3 **Serum antibody responses against lysate, rUreB and rNapA at three weeks post- immunization.** Mice were immunized twice with three weeks interval with 100 μg HS5 lysate plus 5 μg CT, 30 μg rUreB plus 5 μg CT or 30 μg rNapA plus 5 μg CT, respectively. Three weeks after the last immunization blood was collected and serum was prepared from 5 animals of each group. Data are shown as the mean OD_405 nm_ + SD. *** *p* < 0.001. Click here for file
