# Supplementary figures and images for: Immunization with the immunodominant Helicobacter suis urease subunit B induces partial protection against H. suis infection in a mouse model
Source: Vet Res. 2012 Oct 26;43(1):72. doi: 10.1186/1297-9716-43-72 (PMC3542004; doi:10.1186/1297-9716-43-72)

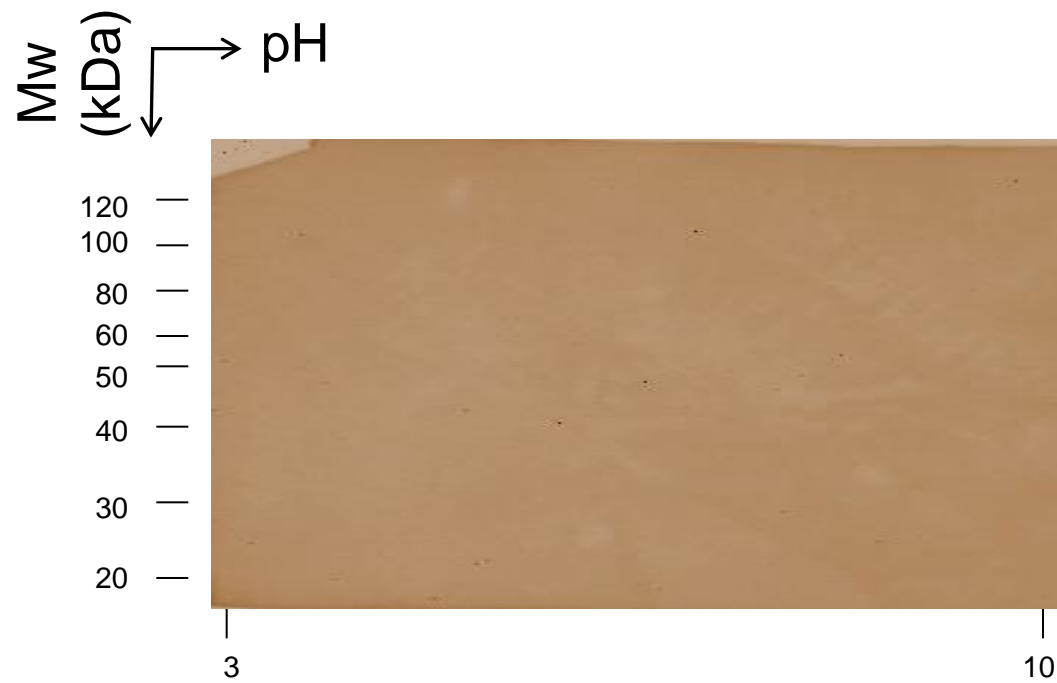

Supplement: Additional file 1 — Immunodetection of a 2D-Western blot with a pool of control sera from H. suis -negative mice. 100 μg of H. suis total protein extract was separated by 2D-electrophoresis using linear pH3 to10 gradient in the first dimension and 10% SDS-PAGE in the second dimension. After transfer of the proteins onto a nitrocellulose membrane, the 2D-immunoblot was analyzed by reacting with a pool of control sera from 10 H. suis-negative mice. No specific immunoreactive protein spots were detected. [file 1297-9716-43-72-S1.pdf]

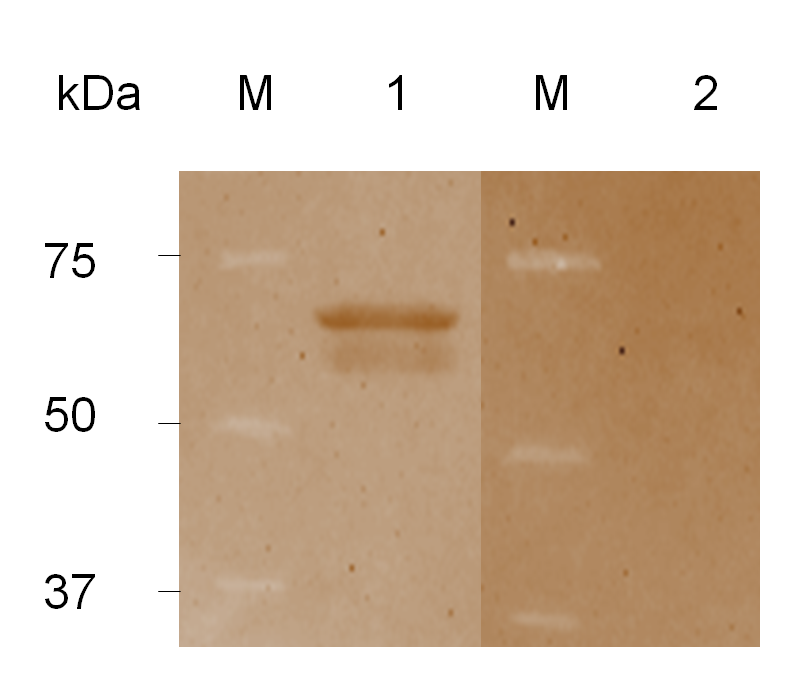

Supplement: Additional file 2 — 1D-PAGE immunoblotting of rUreB. M: Protein marker. Lane 1 and 2: 10 μg rUreB separated on 10% TrisHCl SDS-PAGE and immunoblotted with serum of mice 3 weeks after immunization with H. suis whole-cell lysate (1) or with serum of H. suis-infected mice at four weeks post-infection (2). Both sera consisted of a pool of 10 animals. Only in serum of immunized animals (lane 1) immunoreactivity against rUreB is seen as a ~ 63 kDa band. [file 1297-9716-43-72-S2.tiff]

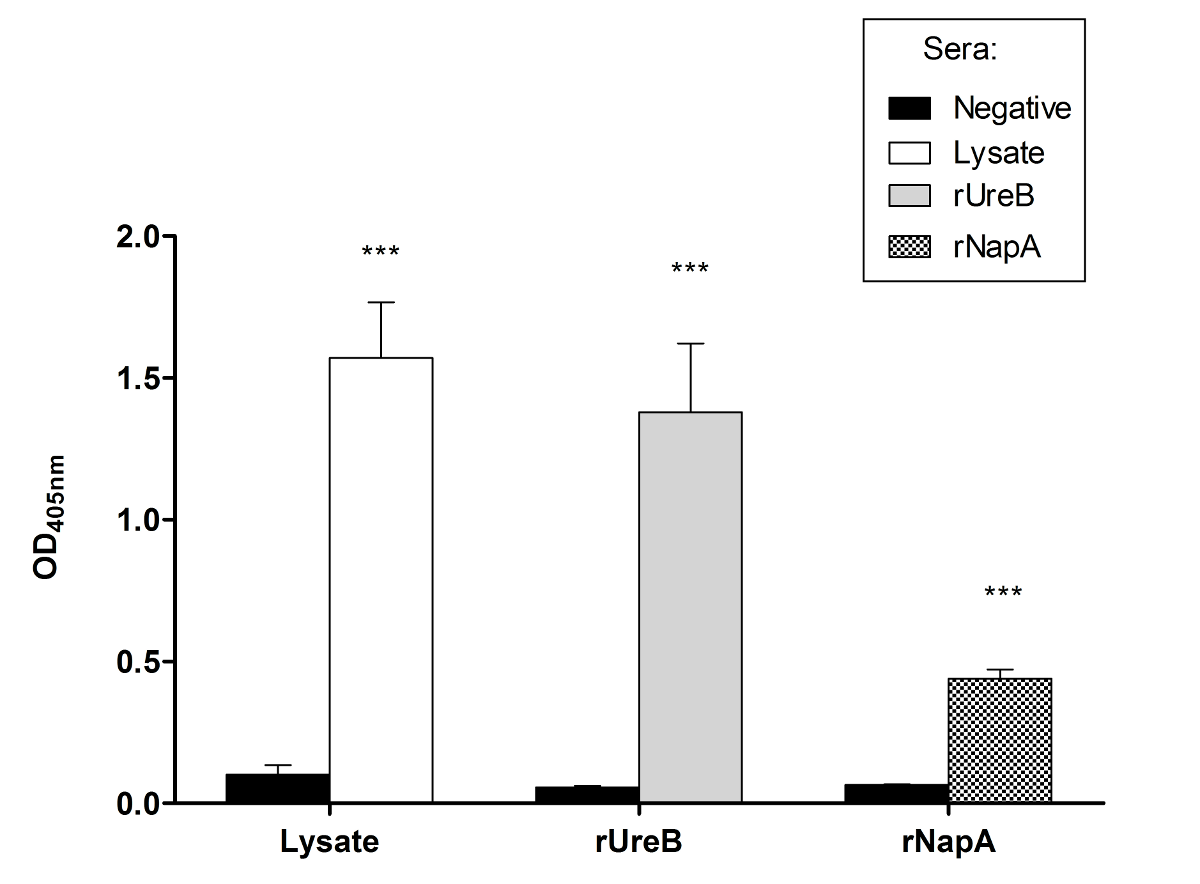

Supplement: Additional file 3 — Serum antibody responses against lysate, rUreB and rNapA at three weeks post- immunization. Mice were immunized twice with three weeks interval with 100 μg HS5 lysate plus 5 μg CT, 30 μg rUreB plus 5 μg CT or 30 μg rNapA plus 5 μg CT, respectively. Three weeks after the last immunization blood was collected and serum was prepared from 5 animals of each group. Data are shown as the mean OD405 nm + SD. *** p < 0.001. [file 1297-9716-43-72-S3.tiff]
